# Differences and similarities between mothers and fathers of premature children: a qualitative study of parents’ coping experiences in a neonatal intensive care unit

**DOI:** 10.1186/s12887-016-0631-9

**Published:** 2016-07-15

**Authors:** I. H. Hagen, V. C. Iversen, M. F. Svindseth

**Affiliations:** NTNU, Norwegian University of Science and Technology in Aalesund, Postbox 1517, 6025 Aalesund, Norway; NTNU, Norwegian University of Science and Technology in Trondheim Institute of neuroscience, Postbox 8905, Medisinsk teknisk forskningssenter, 7491 Trondheim, Norway

**Keywords:** Neonatal, Premature birth, Parents’ experience, Coping, NICU

## Abstract

**Background:**

The aim of this study was to explore and describe the coping experiences of parents to children admitted to a neonatal unit.

**Methods:**

A qualitative research approach was chosen, using in-depth interviews with eight fathers and eight mothers.

**Results:**

The main findings were that parents with previous complicated births had more difficulties in coping compared to those parents with no experience with complications. Coping seemed easier where parents’ opinions were heard regarding their baby’s care and when both parents were present in the neonatal intensive care unit (NICU). The main similarities between mothers and fathers were the reluctance to speak their opinions on childcare, and both experienced a sense of alienation and problems in bonding with the baby. They also needed a limitation on the number of visitors in the NICU. Differences between mothers and fathers were that fathers tried hard to be the strong partner in the relationship, and were more concerned with the mother if she was seriously ill postpartum, while mothers were more concerned for their baby. Mothers’ postpartum period was felt as more stressful if the father was not present, but mothers were also better at welcoming support from the health personnel.

**Conclusion:**

This study highlights the parent’s coping experiences in NICUs. Coping seemed easier where parents’ opinions were heard. Nurses in the NICU should take the former experiences of the parents into consideration when nursing in the NICU and planning for discharge.

## Background

Premature birth is likely to be a huge trauma entailing existential reflections on the possibility of death or handicaps. The infant will be connected to all kinds of scary instruments, and most parents are unfamiliar with the medical language and the various alarms going off [1]. Most of the parents have difficulties to cope with a critically ill, premature child or, worst case, unexpected infant death. The lack of preparation for parenthood, the hospitalization itself, along with grief and isolation all contribute to a very difficult emotional situation for mothers and fathers [2]. The parents certainly must feel insecure in the situation that has suddenly appeared. Different experiences in the neonatal intensive care unit (NICU), which could be difficult to understand, will also affect the parents’ coping in taking care of oneself, the rest of the family, and the infant [3].

Every year approximately 6000 new-borns are admitted to a neonatal intensive care unit in Norway, due to critical illness or suspicion of critical illness. The number of individuals in need of advanced health care is quite large, approximately 10 % of all new-borns [4]. The Norwegian Health Directorate (2007) published guidelines on how to treat premature children in Norwegian NICUs, focusing a great deal on enabling the parents to feel as safe as possible in order to optimize coping [5]. The parents are by Norwegian law [6–8] given the right to be with their child around the clock, to receive proper information and guidance, and to take part in decisions affecting their child.

Several studies report giving birth to a premature child as very stressful and raise many different emotions in both the mother and the father. Parents are exposed to a mixture of emotions like anxiety, worries, fear, guilt, and helplessness associated with a premature birth [9–13]. Just a few studies describe the parents’ coping mechanisms in a premature birth situation [14–17]. Huges et al. [15] found that mothers use different coping mechanisms compared to fathers. Fathers seemed to get most of their support from the nurses and health personnel and were more problem-solving oriented as a coping strategy. By contrast, mothers seemed to get most of their support from their spouses and coped by focusing on what seemed positive in the situation. Mundy [18] reported that parents ranked assurance needs as most important, such as being informed, when away from the NICU, about important changes in the infant’s condition and being assured that the infant is given the best possible care. Parents reported comfortable furniture and support groups as less important in coping as inpatients in a NICU.

The process of connecting emotionally with the child is more difficult when giving birth to a premature child. Fegran et al. [19] examined six mothers and six fathers. The connection process was different across gender lines, as the fathers experienced the time in the NICU as the start of a relationship with the child, while the mothers experienced more helplessness. Jackson et al. [20] found that mothers were more reluctant to prepare for the future, interpreted as fear of their infant passing away. The fathers reported a strong feeling of guilt because they also had to take care of their work and other family members, and were therefore more absent from the newborn. Feeley et al. found that both parents reported similar levels of anxiety and perceived helpfulness from the support they received and were equally sensitive and responsive in interactions with their infants at 9 months of age. Fathers’ self-efficacy was significantly lower than mothers’, although the fathers reported more received support than mothers [21].

There is a lack of research on parents’ coping strategies when parenting a premature newborn admitted to the NICU [22]. The purpose of this research was to describe how mothers and fathers perceived giving birth to a premature infant and coping with parenting their newborn in the NICU.

Our research questions are as follows:

1. Which factors affect parents’ coping experience in the NICU?
2. Which similarities and differences can be identified between fathers’ and mothers’ coping experiences?

## Methods

### Study setting

The study took place in a NICU in a hospital located in the west of Norway. The NICU holds 14 beds and treats patients with medical and minor surgical diseases. Approximately 300 patients are admitted to the NICU each year, of which approximately 20 % are readmitted from their homes. The unit treats patients with a gestation age of ≥ 24 weeks and up to newborns aged three months. On rare occasions treatment may include patients with a gestation age of < 24 weeks. The NICU admits newborns born with a disease or damage and in risk of developing diseases due to their premature birth. Most of the newborns at the NICU are in need of medical treatment and technical support such as incubator or respirator treatment due to breathing problems or immaturity. As with most NICUs, most treatment in this unit entails monitoring and observing the newborns, to respond with proper treatment in acute situations that often occur with premature babies.

The unit has one large room for incubators and most of the parents/families, and one separate room for isolation purposes. There is a reception station in the large room. Parents can be with their children during the whole admission. Parents are also offered a room in a building next to the NICU. In the NICU parents also have access to a recreation room with television and fridge. Meals are served in the hospital café.

### Population/informants

To be included in the study, both parents had to be willing to be interviewed, had to speak Norwegian, and had to be older than 18 years of age. Both parents should be interviewed one to six months after discharge to ensure that none of the parents are in immediate crisis or too distorted on memory of events. The child should have a gestation age of less than 32 weeks at birth. A letter with study information and consent to participate was sent to the parents’ home address. After three weeks the clinical nurse manager of the intensive care unit phoned the unresponsive parents to ask if they were interested in participating.. Eight mothers and their spouses were recruited over a six-month period. Data were collected between February and September 2008 by the lead author.

### Preparation for data collection

Early on in the study the first author spent time reflexively developing an understanding of how the researchers’ backgrounds as neonatal nurses could affect the research. Research in the researcher’s own field of expertise may be somewhat biased due to the preunderstanding of phenomena and the inability to identify new dimensions of the research topics. On the other hand, preunderstanding may also be positive by allowing for an easier understanding of what the informants say and for a better ability to ask relevant follow-up questions during the interviews.

### The interviews

A semi-structured, theory inspired interview guide was constructed to elicit answers that best fit our research questions. Pre-test interviews were performed, and some of the questions in the interview guide were changed to make them more understandable. Adjustments were also made to the interviewer’s role, which gave more time for the informants to answer the questions. Mothers and fathers were interviewed separately to avoid influencing each other. All respondents were interviewed from one to six months after discharge from the NICU although the majority of the interview were conducted about three months after discharge. Most of the interviews were conducted in the respondents’ home or workplace. A few took place in a quiet room in the hospital at a time when the infant was scheduled for medical follow-up. Each interview lasted between 40 and 90 min. Data collection ended with data saturation – i.e. when no new information emerged from the interviews. All interviews were audio recorded and the first author wrote field notes. Questions pertained to time phases of the admission, stays and discharge, and the experiences with different health personnel, the environment, and the care of the child, siblings, and the family as a whole. At the start of each interview the participant was given the opportunity to ask questions prior to signing the informed consent form.

### Preparing the data for analysis

The author transcribed audiotapes verbatim as soon as possible after the interviews. Interpretive summaries and coding of individual statements from the data allowed identification of themes. The analysis followed established conventions for qualitative research and was underpinned by Malteruds’ [23] understanding of phenomenological systematic text condensation, as inspired by Giorgi [24].

### Data analysis

The analysis started with re-reading the transcripts to achieve an overview of the informants’ narratives. The predefined themes in the semi-structured interview guide steered the choice of dimensions, although the researcher was also open to new knowledge or deleting dimensions according to the narratives. Each couple was initially analysed at the same time to find similarities and differences. The analysis thereafter was searching for similarities and differences gender wise in the whole group of informants, comparing men and women by systematic decontextualization. Text condensation was also performed with the keyword “coping”, and the material was analysed by putting all the gender statements into a table with the same coding as the previous analysis step. Fifteen different codes were revealed, and the table provided differences in experiences between gender in a visual manner that gave an overview to the answers to the research questions. The last step of analysis was to recontextualize to ensure that no important statements were missing. One independent researcher went through the transcribed material and agreed to the findings and identified dimensions.

## Results and discussion

As shown in Table 1, the mean age of the mothers was 34 years, while the fathers was 36 years. The infants’ mean age was GA 30, and mean birthweight was 1500 g. Mean weeks of hospitalization were nine.

**Table 1:** Characteristics of the parents completing the interview

	Range	Mean	St. Dev.
Mothers - age	22–41 years	34	6,3
Fathers - age	25–45 years	36	7,0
Infant gestation age	27–32 weeks	30.0	1.4
Infants- weight	870–1780 gr	1500	314.7
Hospitalization	3–20 weeks	9.0	5.7

The themes that emerged from the data were: approaching the baby, being a companion, chaotic emotions, and parenting role

### Chaotic emotions

Both parents initially experienced the stay in the NICU as unreal and full of different emotions. Overall statements from the parents entailed a sense of something always happening and the feeling of being busy. Moreover, the experience of not filling their role as a parent seemed to be overwhelming for the parents, in other words a lack of coping in the situation.

Promoting a good parental experience is of utmost importance to the families experiencing a crisis such as having a premature baby. They report fear of losing their baby:

*I was full of stress and I was scared. There is so much hope and joy and in the next moment only sadness. And then there is the feeling of not having control. It is like being in a parallel world. You are on the highest level of anxiety all the time with no possibility to live the usual normal life.* (Mother 28 yrs, GA: 30)

Chaotic thoughts and feeling unable to take care of the baby were explained like the following:

*I was happy to leave all responsibilities to the health personnel. All the time my baby was in the incubator, I was not strong enough, I had more than enough by handling my own existence in the situation* (Mother 40 yrs, GA: 30).

Other studies also documented the commonality of chaotic emotions in the role of being a parent to a premature child [9–13].

Both parents experienced the first period after premature birth as unreal. They felt as if they were part of an audience looking at the situation from an almost outsider position. They experienced contradictory emotions like joy, sorrow, hope, or despair. They used words like surrealistic, being in a parallel world/existence, being in a protected world, or in a vacuum*:*

*“[…] but the experience was surrealistic. You witness your child developing through the window of the incubator. You witness something that you should not witness”* (Mother 35 yrs, GA: 29).

When a parent is in a situation that lacks a sense of reality, it is difficult to understand and come to grips with what is communicated, resulting in an inability to cope The above mentioned mother still managed to have a sense of coherence and obviously reported confidence in health personnel, thus giving her the opportunity to focus on herself. Both parents reported sensing a lack of reality, like being in a parallel world and having a spectator role, looking into “something” from the outside. The fathers in particular talked much about feeling alienated. Several fathers told about their inability to think clearly during the NICU admission and the time afterwards. They felt like spectators and acted automatically.

*I had no time to think or reflect, I was like a spectator* (Father 25 yrs, GA: 30).

This finding is important to note. In further planning of health care in NICUs, the fathers should be more involved in tasks that will allow them to cope in relation to their newborn. Hugill et al. [25] reported that most fathers, when first meeting their baby, felt a provoking feeling of serious loss of emotional control. Their narratives also described the intense interior distress they encountered. They had often tried to react by hiding their emotions behind stereotypical behaviours in order to protect themselves from further pain. These finding are in concordance with this study. As one of the fathers in our study described;

*I have to admit that some of the days, when I was driving home from the NICU, the ‘raindrops’ were not just on the outside of the car* (Father 43 yrs, GA: 27).

He wanted to be strong in front of his wife and child, but his emotions overwhelmed him as soon as he was alone, although probably not specific to fathers of premature children admitted to the NICU or even healthy newborns.

Even if the parents experienced the situation as unreal and surrealistic, most of them also experienced being in both a scary but also a safe situation. The couples that had previously experienced ordinary pregnancy and birth or had never given birth (three couples), had limited knowledge on how to cope and felt somewhat differently from those parents with former experiences from an NICU or problems in pregnancy. The couples with previous experience of complicated pregnancies or births (five couples) wanted to talk about earlier experiences and compare those with the present pregnancy. The parents who had past experience with giving birth to a premature baby (three of the five couples) were especially occupied with comparing past and current pregnancy and birth. Our informants stated that previous birth experiences definitely could colour the coping in later pregnancies and births. Both parents were afraid that something dramatic would happen to their baby, as they had previously experienced that life is fragile with small margins between life and death in the NICU. Even if our informants reported negative emotions due to previous experiences, we cannot exclude that other parents could benefit from earlier experiences, having more knowledge and coping mechanisms. After discharge they bought apnoea detection bed to be sure they would know if their baby stopped breathing. Nurses in the NICU should take the former experiences of the parents into consideration when planning for discharge to homes. Parents without former experiences with premature birth did not focus on the catastrophic thinking and coping compared to parents with former experiences. One of the mothers lost a child in GA 30. When she was giving birth, she immediately thought:

*I asked him (the unborn baby), ‘Are you also going to die?’* (Mother 40 yrs, GA: 30).

Another mother put it this way:

*The entire pregnancy was a nightmare for the both of us (mother and father). When it appeared that this birth would also be premature, we were convinced that the whole disaster would repeat itself. Even if all went well, I could not understand that before we were safe back home with our baby in our own home* (Mother 28 yrs, GA: 30).

These experiences tell us that former pregnancies and births must be taken into consideration in order to provide optimal health care. We have not found previous studies to support this finding.

### Approaching the baby

Even if our informants report difficulties in relating to their newborn, the mothers and fathers seem to instinctively recognize the needs of the other part, and together they manage to cope with the new situation. When the mothers are too weak to cope, it seems as if the fathers take responsibility, and vice versa, when handling their newborn. All informants were content with the treatment they received in the NICU. They were impressed with the high level of competence and impressed with the individual treatment of each baby. Some parents had very high expectations of themselves and tried very hard to live up to those. Some also founded that they did not manage to meet their own expectations when being a mother or father. They needed time to accept the situation and relate to their baby. When the worn out parents, possibly in a state of emotional instability, were “pushed” to take part in handling their newborn, they felt discomfort and the inability to cope.

*I did not bond to the child, but still I felt that I would bond in the future. It seemed more like the premature baby would be my child after a while. She (the baby) was like a foetus […] Quite unreal […,,]* (Father 43 yrs, GA: 27).

The reason can be that parents did not expect the baby so early and that they have not developed the maturity due to the early date of birth. They implicitly suggested that they needed more time to bond with the baby. This is somewhat contradictory to the finding of Fegran [19]. In her study the fathers were ready to involve themselves immediately, while the mothers needed more time.

Health personnel are in an ideal position to influence the parents and challenge them to do what is good for their baby. Still, to achieve compliance the parents also need to trust the health personnel, and trust can be the ultimate factor for how the parents achieve an adequate coping feeling in an NICU.

For the parents, it appears to be very important that the health personnel accepts and understands the various emotions in a timely manner.

*They (health personnel) were right when they told me that I would bond with the baby when I was emotionally ready* (Mother 40 yrs, GA: 30).

Stern [26] theorizes on the rituals of bonding and that the natural processes are disturbed in premature births. The picture in the parents’ mind will be disrupted, as reality is quite different from their expectations. Parents are afraid of harming their baby and are torn between emotions like attraction versus fear, sometimes even disgust and always helplessness. These emotions were common among our informants as well.

Most of the parents spoke about the kangaroo method, which has been reported as important in the bonding process between parents and babies. Although we had no questions in the semi-structured interview guide about kangaroo care, all parents reported on the importance of this method. Kangaroo care is a technique practiced on newborns, usually preterm, infant where an adult holds the infant skin-to-skin. This technique has in some study shown positive health effects [27]. The parents reported that the NICU provided good information and environmental adjustment for practising kangaroo care. Some fathers found the kangaroo method a bit frightening, but at the same time they reported that the method provided a stronger attachment to the baby compared to the usual way of being close to their newborns:

*That’s when you feel that you are responsible for the baby, for the first time. You realize that it is your daughter* (Father 41 yrs, GA: 30).

One of the mothers described the kangaroo method:

*….when she was born and they placed the baby on my chest […] it was like having my heart put in place. I realized that she was mine* (Mother 41 yrs, GA: 27).

All parents reported the importance of kangaroo care but disagreed on when and how long to use the method each day.

*We were almost forced to have the baby lying on our stomach. The baby lies there and we observe lack of oxygen in the system. We had to stimulate to make her breathe again […] Sometimes I believe that I should not have taken the baby out for kangaroo treatment, or put her back into the incubator before I did. I sat, with the baby on my stomach, I was sweating […] and looking at that machines and listening to the alarms going off and this made me very anxious. I do not believe that the kangaroo method is only beneficial, but it is not easy for us to speak our mind (Father 41 yrs, GA: 30).*

Some parents were reluctant to tell the health personnel, probably due to their vulnerable position and because they felt that the health personnel had the proper knowledge to advise them. They wanted to do the best for their newborns, although they had their critical opinions, especially about the time and length of time they were supposed to provide kangaroo care. It is possible that the above also can negatively influence the parents’ coping. Health personnel should be advised to listen to the parents and collect data on each of the parents according to when they feel comfortable by using the kangaroo method and how long time they can transfer calmness to their newborns. The time after birth is an ongoing process with emotions like responsibility, trust, alienation, powerlessness, and surrealism [22, 23]. These emotions are also described by our informants and show that the kangaroo method is perceived as both positive and negative. By learning more, health personnel can adjust the method so it will suit the individual person in NICUs.

### Parenting role

The role of parenting contains activities and attitudes towards the baby, but it also includes the role that both parents fill within the family as a whole. In cases where the mothers were seriously ill postpartum, the fathers were more concerned for the mother’s recovery. The explanation might be that when a child is premature, fathers seem to have difficulties in understanding that he has become a father [26]. In addition, when the mother is seriously ill, it is easy to understand that his main focus will be on her in the first period after birth. It also seems that fathers need time to adjust emotionally to their role of being a father.

The data suggested that fathers compensate for the mother’s incapacity whilst ill by being more active than usual. The mothers appear to do the same when fathers are absent. Most parents expressed that their focus on cooperation with their partner was important. They helped each other. Ward argued that the most efficient way of making the parents cope was to take good care of their child, along with providing the parents with enough information on the child’s status [17]. Parents in our study felt that they were given good information and training in caring for their baby in the NICU. They also expressed great trust in the NICU’s health personnel. Nevertheless, they also reported “lacking the guts” to speak their mind about their own preferences in coping with caring for their baby and mentioned their fear of negative sanctions if they did not comply with everything they were told. They also reported feeling pressure to fulfil the parenting role perfectly, and many mothers reported lack of coping due to the massive demands they felt. By exploring this topic further, it appeared that the lack of coping the parents felt was mostly due to their own emotions and demands to themselves, rather than anything the health personnel told in words or in non-verbal signals.

*It was the feeling of not being in control. It is like being in another world. You are in high gear all the time. Like when I went to eat, I experienced feeling guilty – the same happened if I went for a walk. I felt that the nurses wanted me around all the time and if I was a bit late, they had already changed her diaper when I came back. You become very attentive to these things [….]* (Mother 28 yrs, GA: 30).

Many of the mothers had very high expectations of themselves and were taught that the parents should always be the main people around their babies. Jackson [20] found that mothers experienced having more responsibility and control of the care and a need to be confirmed as a mother, while fathers described confidence in leaving care to the staff and wanted to find a balance between work and family life. This applies to our study as well. None of the fathers complained of an inability to cope with caring for the babies, but the mothers reported a lack of coping.

### Being a companion

According to Norwegian legislation children have the right to have one of the parents present around the clock. The legislation also delineates different rights according to who is allowed and not allowed to be present in the NICU. The legislation sees the bonding process as very important and provides the parents the opportunity to be active as a family from day one. Most of those parents who were present described feeling stress as well as feeling safe. Having other parents close by in the NICU seemed to contribute to cooperation and safety and thus influencing coping.

*It was ok that the NICU was somewhat crowded. It is good support to have other parents in the same situation close by* (Mother 36 yrs, GA: 31).

The parents reported a need to be on their own as well. At the same time, they felt safe being close to other parents and staff. This supports the findings of Jackson [20], who reported that parents find comfort in being with others in the same situation.

For the mothers, accompanying their child can be perceived as stressful when the fathers are unable to be in the NICU. Our study shows that the mothers who were not accompanied by the fathers had difficulties in coping compared to those who were accompanied by the fathers. It was particularly difficult and stressful when having to spend the night in another building apart from their baby when they were on their own.

*It was very important to be two* (Mother 22 yrs, GA: 30).

Another mentioned that

*the most difficult was when my wife had to be on her own in the hospital. She struggled a lot with that part. I had lots of phone calls with my wife during those nights. It should not be like this. Mother and baby should be together* [Father 45 yrs, GA: 31].

Huges et al. [17] reported that mothers in NICUs cope better when fathers are present.

The NICU philosophy and the trust in staff were essential for parents to cope and manage the critical situation. The Newborn Individualized Developmental Care and Assessment Program (NIDCAP) treatment principles are incorporated in most NICUs in Norway. This principle emphasizes the importance of including the parents in treatment and their baby’s care in an early phase and gradually taking on more and more responsibility for the baby’s care.

All parents in this study experienced trust in the NICU’s staff. They experienced continuous care and being provided with solid information. Many parents also reported being impressed by the high level of knowledge among the NICU’s staff. The high level of knowledge provided a sense of safety and trust in health personnel.

*The NICU was like being in a different world. Personally I felt like being in a womb. Like being in a protected world. All the responsibilities and lifestyle you had outside the NICU were gone* (Mother 40 yrs, GA: 30).

Satisfaction with the treatment and care in total has also been reported in several other studies [28, 29].

Most parents consider the introduction of their baby to family and friends as a huge event in their lives. However, this was not the case for most parents in this study, who were reluctant to welcome visitors.

*I had no need to show my baby because he was not finished yet* (Father, 37 years, GA: 29).

Stern [26] suggests that parents to premature infants do not seem to be ready as parents, compared to those giving birth to full-born babies. At the same time, they needed the close family to understand what they were going through.

*[…] if my family had been here I would not have to explain so thoroughly to make them understand. They would then have understood all the fear we had to go through* (Mother 28 yrs, GA: 29).

This is consistent with findings in other studies [17]. The parents also reported being afraid of infections from the outside world if too many came to visit.

### Summing up

Which factors affect parents’ coping experiences?

- Coping with the present situation was negatively influenced by previous childbirths where the baby had been ill or premature, or passed away.
- The parents’ coping was positively influenced when nurses and health personnel considered the parents’ opinions and needs regarding caring for their baby as important..
- The fathers’ presence seemed to positively contribute to coping for both parents.

Differences and similarities between mothers and fathers:

Similarities:

- Both mothers and fathers appeared to trust the health personnel, but they were also somewhat reluctant to give their opinions regarding their baby’s care.
- Both had a sense of alienation regarding everything that happens postpartum.
- Both experienced problems regarding bonding with their baby.
- Both felt the need to protect themselves from visitors and other baby’s next of kin.

Differences:

- The father often tried to fit the role of the strong postpartum partner, and he hid his own needs.
- In the cases where the mothers were seriously ill postpartum, the fathers were more concerned about the mother than the baby, whereas the mothers were most concerned about the baby.
- Mothers experienced more stress when accompanying the baby in the NICU without having the fathers present.
- When compared to the fathers, it seemed like the mothers experienced more support from the other parents in the NICU.

#### Strengths and limitations

The first author conducted the interviews and the transcriptions. Emotional expressions like tears, anger or other emotional issues were noted in order to provide information on the atmosphere of the interviews as well as hidden meanings of the words. This is considered a strength. A limitation is that the informants did not read and comment the transcripts. Another limitation could appear from the pre-understanding of the interviewer, but this could also be seen as an strength since the interviewer had the ability to ask the right questions due to long experience in NICU. The informants and their infants were all in the recovery phase when interviewed. Other parents with infants with sequels could have reported other experiences with the NICU. Issues could be interpreted differently than intended by the informant although we do not consider this as very probable due to the thoroughly transcripts and open-ended questions. Given the methodology of the study results could not be generalized.

## Conclusion

The main findings were that parents with previous complicated births found coping more difficult compared to those parents with no prior experience of complications in childbirth. Nurses in the NICU should take the former experiences of the parents into consideration when nursing in the NICU and planning for discharge. Coping also seemed easier where parents’ opinions were heard regarding care of their baby and when both parents were present in the NICU. The main similarities between mothers and fathers were the reluctance to speak their opinion on childcare, a shared sense of alienation, and problems with bonding with the baby. Health personnel should be advised to listen to the parents and collect data on each of the parent according to when they feel comfortable by taking part in care and using the kangaroo method.

Differences between mothers and fathers were that fathers tried hard to be the strong part in the relationship. Fathers often felt as spectators and tried to fit the role of the strong partner, and often hid his own needs.

Further research on emotional challenges and coping mechanisms in fathers and mothers having a premature child is recommended.

## Abbreviations

NICU, Neonatal intensive care unit
